# Contemporary Concise Review 2025: Interstitial Lung Disease

**DOI:** 10.1002/resp.70309

**Published:** 2026-09-07

**Authors:** Tonia Magrì, Luca Richeldi

**Affiliations:** ^1^ Università Cattolica del Sacro Cuore Rome Italy; ^2^ Fondazione Policlinico Universitario A. Gemelli IRCCS Rome Italy

**Keywords:** early diagnosis, interstitial lung diseases, molecular biomarkers, multidisciplinary diagnosis, precision medicine, progressive pulmonary fibrosis

## Abstract

- Multidisciplinary discussion remains the cornerstone of ILD diagnosis and management, with recent advances further strengthening the integration of clinical, radiological, pathological, and molecular information.
- Updated ILD nomenclature and classification better align disease terminology with underlying morphology and pathobiology.
- Growing emphasis is being placed on the early detection of ILD and on identifying individuals at high risk of progression among those with interstitial lung abnormalities (ILAs).
- Emerging multi‐omic biomarkers and quantitative imaging techniques are enhancing prognostic stratification and may support future precision medicine approaches.
- Novel antifibrotic therapies and targeted treatments are expanding therapeutic options beyond IPF, although important unmet needs remain regarding patient selection, treatment response, and disease modification.
- The integration of clinical, radiological, functional, and molecular information will be fundamental to optimize individualized management and improve long‐term outcomes in patients with ILDs.

## 1 Introduction

Interstitial lung diseases (ILDs) encompass a broad and heterogeneous spectrum of respiratory disorders of diverse etiologies, characterized by varying degrees of inflammation and/or fibrosis involving lung parenchyma and, occasionally, airways. While most individual ILDs meet the epidemiological criteria for rare diseases, collectively they impose a considerable clinical and societal burden. Recent epidemiological evidence indicates that ILD incidence ranges from 1 to 31.5 cases per 100,000 person‐years, with reported prevalence estimates varying between 6.3 and 71 cases per 100,000 individuals [1]. Idiopathic pulmonary fibrosis (IPF) is the prototypical and most common fibrotic ILD, predominantly affecting men older than 60 years. IPF is a chronic, progressive disease characterized by relentless and irreversible deposition of extracellular matrix within the lung parenchyma, leading to progressive distortion of lung architecture. This process results in impaired gas exchange, declining lung function, worsening health‐related quality of life (HRQOL), and ultimately respiratory failure and premature death. Similar to IPF, several other ILDs may exhibit a progressive fibrosing phenotype, sharing comparable disease trajectories and poor outcomes [2]. To better identify these patients, the latest ATS/ERS/JRS/ALAT clinical practice guidelines introduced the concept of Progressive Pulmonary Fibrosis (PPF), encompassing fibrotic ILDs other than IPF that demonstrate disease progression despite appropriate management [3].

This contemporary review highlights the most impactful evidence published over the past year in the field of ILD, with particular emphasis on emerging classification approaches, advances in early diagnosis and biomarker research, novel therapeutic strategies, and persisting unmet needs.

## 2 Classification

**Summary**

- Updated ILD classification distinguishes morphological patterns from the final multidisciplinary diagnosis.
- ILDs are classified into interstitial, alveolar filling, and other (combined or unclassifiable) patterns.
- Updated nomenclature introduces bronchiolocentric interstitial pneumonia (BIP) as a distinct morphological pattern.

Over the past decades, several international multidisciplinary classification systems for ILDs have been developed and periodically updated. These continuous revisions reflect the growing understanding of ILD pathobiology and highlight the critical importance of accurate diagnosis and classification, given the substantial differences in prognosis, treatment approaches, and monitoring strategies across ILD subtypes.

Earlier classification systems primarily focused on idiopathic interstitial pneumonias (IIPs) and relied on clinical‐radiological‐pathological correlations to define distinct disease entities. Over time, the increasing recognition of overlapping phenotypes, unclassifiable forms, and shared biological and clinical features across different ILDs has progressively shifted the field toward a more integrated classification framework [4, 5].

The latest international classification embodies this paradigm shift by clearly distinguishing morphological patterns from the final multidisciplinary diagnosis, which is established through the integration of clinical, radiological, and pathological data [6]. This distinction acknowledges the biological complexity of ILDs and cautions against equating a radiological or histopathological pattern with a specific disease entity.

Accordingly, the diagnostic process is rarely straightforward. Rather, modern ILD diagnosis should be regarded as a dynamic multidisciplinary process integrating phenotypic and biological information to continuously refine diagnosis, prognostic assessment, and treatment decisions throughout the disease course (Figure 1).

**FIGURE 1:**
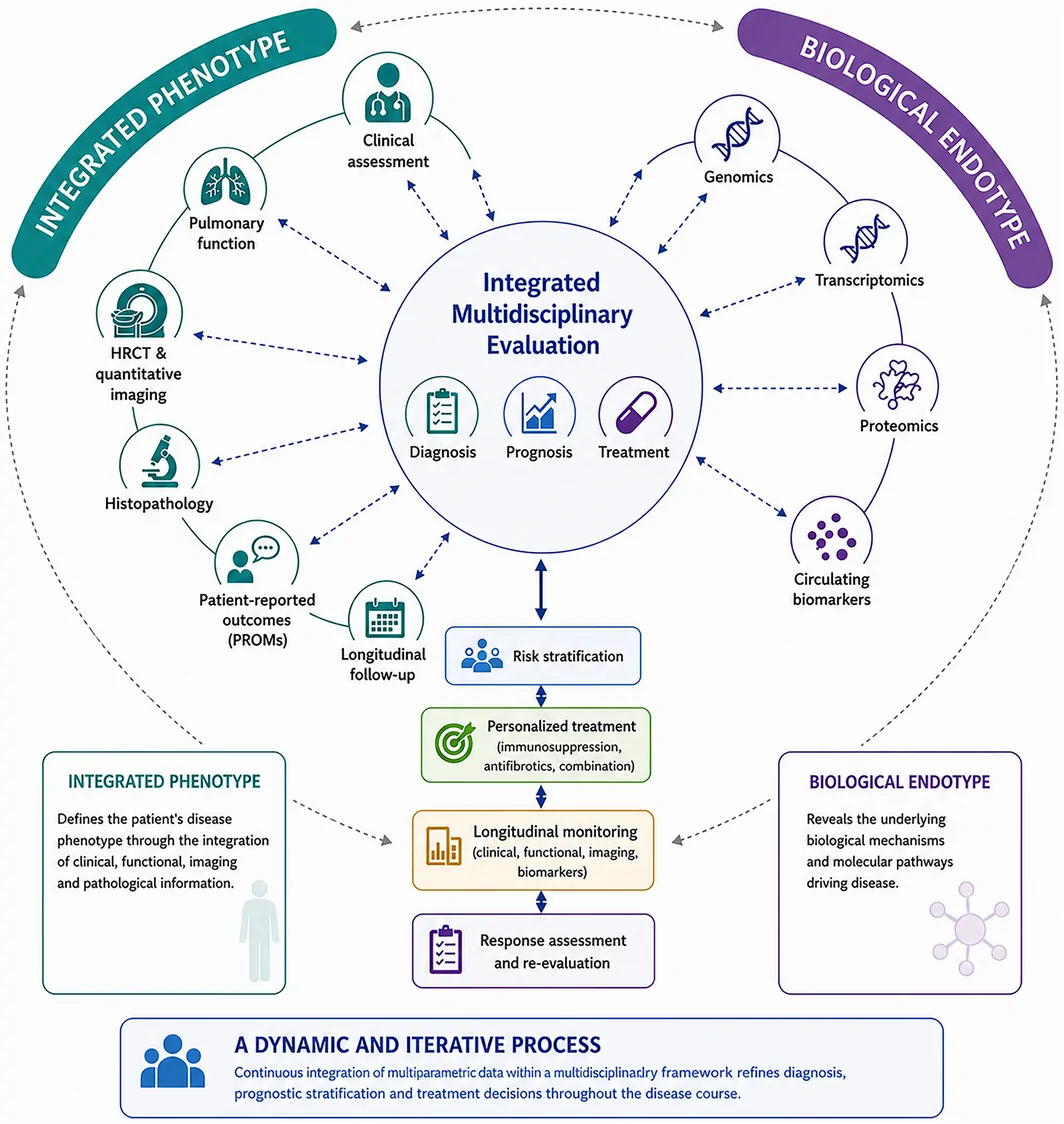
Dynamic multidisciplinary framework for integrated ILD diagnosis and management. Modern ILD management is a dynamic and iterative process in which phenotypic (clinical, functional, radiological and pathological) and biological (genomic, transcriptomic, proteomic and circulating biomarkers) information are continuously integrated within a multidisciplinary framework to refine diagnosis, prognostic stratification and treatment decisions throughout the disease course.

Consistent with this approach, Ryerson et al. recommend that the level of diagnostic confidence be reported alongside the final diagnosis and any relevant differential diagnoses. This approach acknowledges that diagnostic certainty exists along a continuum rather than as a binary construct and promotes greater transparency in multidisciplinary decision‐making [6].

Another major advance introduced by the updated classification is the reorganization of morphological patterns into three overarching categories: interstitial, alveolar filling, and other (combined or unclassifiable) patterns, each encompassing distinct radiological and histopathological entities, as summarized in Figure 2.

**FIGURE 2:**
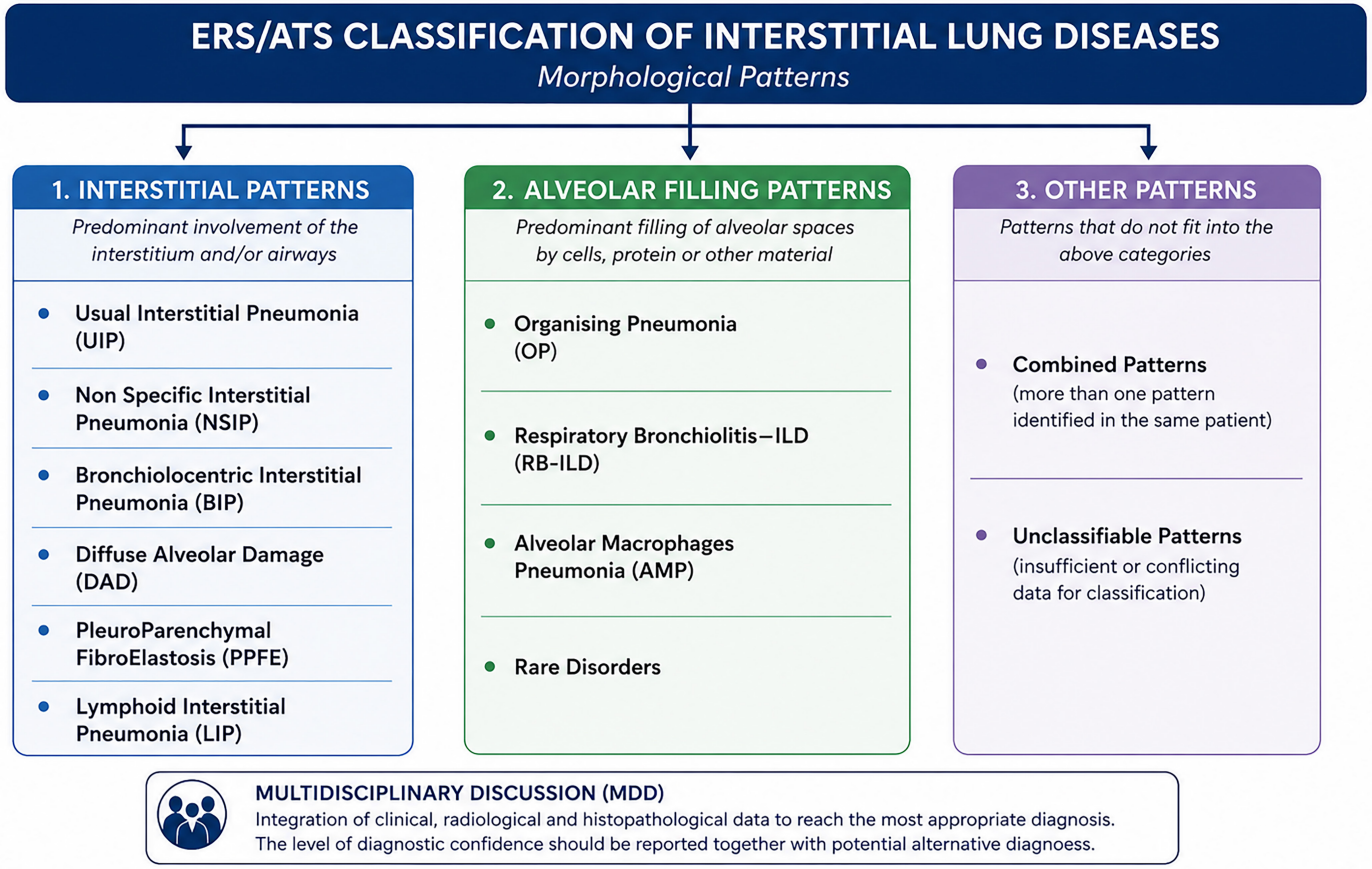
Three Major Morphological Patterns in the Updated ERS/ATS Classification of ILDs The updated ERS/ATS classification groups ILD morphological patterns into three major categories: Interstitial patterns [usual interstitial pneumonia (UIP), non‐specific interstitial pneumonia (NSIP), bronchiolocentric interstitial pneumonia (BIP), diffuse alveolar damage (DAD), pleuroparenchymal fibroelastosis (PPFE) and lymphoid interstitial pneumonia (LIP)]; alveolar filling patterns [organizing pneumonia (OP), respiratory bronchiolitis‐associated ILD (RB‐ILD), alveolar macrophage pneumonia (AMP) and rare disorders]; and other patterns (combined and unclassifiable) [6].

In parallel, ILD nomenclature has been revised to better align terminology with underlying morphology and pathobiology. Notable examples include the introduction of bronchiolocentric interstitial pneumonia (BIP), the replacement of *acute interstitial pneumonia (AIP)* with diffuse alveolar damage (DAD), and the proposed substitution of the historically misleading term *desquamative interstitial pneumonia (DIP)* with alveolar macrophage pneumonia (AMP).

Among these changes, the recognition of the BIP pattern has generated the greatest debate. This pattern was introduced to better characterize ILDs with a bronchiolocentric distribution of abnormalities and to fill the diagnostic gap for radiological patterns suggestive of Hypersensitivity Pneumonia (HP) beyond a consistent clinical context.

The inclusion of BIP among the ‘major’ ILD patterns together with the proposal of idiopathic BIP as a provisional (working) diagnosis has stimulated considerable discussion within the scientific community.

Indeed, high‐resolution computed tomography (HRCT) findings, as well as histopathological features typical of the BIP pattern outside the context of HP, remain poorly characterized. According to Raghu et al., substituting BIP for HP may reduce the relentless pursuit of a potential environmental exposure in some patients, potentially modifying the disease trajectory [7].

Overall, the standardization of ILD nomenclature represents a crucial step toward disentangling the remarkable heterogeneity of these disorders and fostering a deeper understanding of their underlying biology. The importance of adopting a shared terminology across the ILD community has been further reinforced by the recent Fleischner Society consensus statement, which provides standardized definitions and terminology for ILD diagnosis, multidisciplinary communication, and future research [8].

## 3 Early Diagnosis of ILD

**Summary**

- ILA has evolved from an incidental radiological finding to a target for systematic screening of high‐risk individuals.
- Current recommendations identify specific populations who may benefit from ILD screening.
- Current management focuses on identifying individuals at risk of progression through longitudinal surveillance and risk stratification.

There is growing interest in the early detection of ILD, driven by the opportunity to identify patients before clinically significant disease development and ultimately improve patient outcomes.

Interstitial lung abnormalities (ILAs) are non‐dependent bilateral parenchymal abnormalities detected on computed‐tomography (CT), involving at least 5% of a lung zone in the absence of respiratory symptoms, lung function impairment, and evidence of disease progression [9].

The original definition of ILA has recently evolved from an incidental radiological finding [10] in individuals not suspected of having ILD to a concept that supports the systematic assessment of high‐risk individuals. According to the ATS Clinical Statement, the recommended screening population includes smokers undergoing lung cancer screening, patients with connective tissue diseases (CTDs), and first‐degree relatives of patients with familial pulmonary fibrosis (FPF); whereas no recommendation is currently made for chest CT in first‐degree relatives of patients with sporadic IPF due to insufficient evidence [9]. Similarly, ERS/EULAR CTD‐ILDs guidelines recommend screening for ILD in all patients affected by systemic Sclerosis (SSc) and mixed connective tissue disease (MCTD), as well as patients with other CTDs and risk factors [11].

The boundary between ILA and early ILD is often indistinct, as these entities may represent different points along the same disease continuum rather than distinct diagnostic categories. The main clinical challenge is not the recognition of ILAs per se, but rather the identification of those individuals at risk of progression and those who already harbour early ILD [12]. Indeed, although many ILAs remain stable over time, a substantial proportion progress to clinically overt fibrotic ILD, particularly in the presence of fibrotic features, subpleural distribution, definite fibrosis on CT, and more extensive radiological involvement [13]. Additional risk factors associated with ILA progression are older age, smoking history, the MUC5B promoter variant, shorter telomere length, connective tissue disease, and family history of pulmonary fibrosis.

The greatest opportunity lies in identifying individuals who are progressing towards clinically relevant ILD, thereby enabling timely intervention before irreversible disease develops. Consequently, the clinical approach has shifted from the simple detection of ILAs to risk stratification, aimed at distinguishing benign radiological abnormalities from early fibrotic lung disease.

In individuals with ILAs considered at high risk of progression, longitudinal clinical, functional, and radiological surveillance is recommended, with repeat CT imaging after approximately every 12 months. In contrast, for low‐risk individuals, a repeat CT scan every 2–3 years is generally considered appropriate [9].

Although no specific pharmacological treatment is currently available for ILAs, a randomized clinical trial (NCT07201922) is currently evaluating the safety and efficacy of nerandomilast in individuals with ILAs and a family history of pulmonary fibrosis, potentially paving the way for future disease‐modifying therapeutic strategies.

## 4 Biomarkers and Molecular Profiling

**Summary**

- Current research aims to identify biomarkers that predict disease progression and treatment response earlier than conventional clinical endpoints.
- Multi‐omic profiling outperforms single biomarkers for prognostic stratification.
- Proteomic and genomic data are increasingly integrated into clinical assessment to support personalized ILD management.

Despite major advances in imaging and multidisciplinary diagnosis, predicting disease behaviour and treatment response remains one of the greatest unmet needs in ILDs. Current monitoring still relies largely on physiological decline and radiological progression, which often require months before clinically meaningful changes manifest. Importantly, the current definition of PPF is inherently retrospective, highlighting the need for early identification of patients at risk of progression [14].

Among circulating biomarkers, matrix metalloproteinase‐7 (MMP‐7) has consistently emerged as one of the most robust predictors of disease progression. In a recent multicentre longitudinal study of antifibrotic‐treated IPF patients, elevated pre‐treatment serum MMP‐7 independently predicted 12‐month disease progression and overall mortality. Importantly, combining MMP‐7 with additional circulating biomarkers further improved prognostic performance, supporting the concept that multi‐biomarker signatures may outperform individual molecules for risk stratification [15].

Beyond prognostic assessment, circulating biomarkers may also provide an earlier indication of treatment response than conventional physiological endpoints, potentially allowing timely therapeutic adjustments before irreversible lung function loss occurs [14].

The field is now moving beyond individual biomarkers toward comprehensive molecular profiling. Unlike genomics and transcriptomics, proteomics provides a functional snapshot of disease activity by capturing dynamic changes in protein expression and biological pathways, thereby offering greater translational potential for biomarker discovery [16].

A large multicentre proteomic study identified 44 circulating proteins independently associated with 3‐year transplant‐free survival in non‐IPF ILDs. Notably, most biomarkers showed similar prognostic performance across different ILD subtypes and substantially overlapped with those previously identified in IPF, suggesting the existence of shared molecular pathways driving progressive fibrosis. Furthermore, proteomic models combining multiple proteins with clinical variables outperformed conventional clinical models, supporting a shift toward biology‐based risk stratification and paving the way for future molecular classification of ILDs [17].

In parallel, genomic medicine is progressively reshaping the clinical management of ILDs. Genetic testing is increasingly integrated into the multidisciplinary evaluation of patients with suspected familial or early‐onset pulmonary fibrosis, particularly to identify pathogenic variants in telomere‐related and surfactant‐related genes. In a recent prospective multicentre study, the implementation of a dedicated genetic multidisciplinary discussion modified therapeutic management in nearly one‐third of patients, highlighting the growing clinical relevance of genomic information for diagnosis, prognostic assessment, and treatment selection [18].

Collectively, these findings support the integration of multi‐omic biomarkers into conventional clinical assessment, with the potential to improve prognostic stratification, predict treatment response, and enable more personalized management of ILDs.

## 5 Imaging Advances

**Summary**

- Quantitative CT provides a more objective and reproducible assessment of fibrotic lung disease than conventional visual HRCT evaluation.
- Imaging biomarkers provide prognostic information beyond conventional HRCT patterns.
- Photon‐counting CT and FAPI‐PET represent promising technologies for early detection, risk stratification, and treatment monitoring.

High‐resolution CT (HRCT) remains the cornerstone of ILD diagnosis and represents one of the three criteria involved in disease progression definition for PPF. However, assessment of radiological progression still relies predominantly on visual interpretation, which is inherently subjective and affected by considerable inter‐reader variability.

To overcome the limitations of visual HRCT assessment, several quantitative CT (qCT) algorithms have been developed, ranging from texture‐based analysis to machine learning and deep‐learning approaches. Among the best‐established quantitative imaging platforms are CALIPER [19], data‐driven texture analysis (DTA) [20], quantitative lung fibrosis (QLF) [21], and AirQuant [22], which enable objective quantification of fibrotic abnormalities, airway morphology and vascular alterations. Several studies have demonstrated that qCT metrics predict lung function decline and mortality with greater reproducibility than conventional visual assessment [23]. However, widespread implementation remains limited by the lack of standardized acquisition protocols, incomplete external validation, restricted availability, and the absence of universally accepted thresholds for clinical decision‐making [23, 24].

Beyond quantitative image analysis, increasing attention has been paid to identifying image biomarkers capable of predicting disease behaviour. Recent evidence indicates that specific baseline CT features carry important prognostic information. In the COPDGene cohort, traction bronchiectasis and a UIP‐spectrum pattern independently predicted progression of interstitial lung abnormalities, supporting their role as early imaging biomarkers of disease evolution [25].

Conversely, Kim et al. demonstrated that patients sharing the same UIP pattern may experience markedly different trajectories according to the underlying ILD subtype, particularly in CTD‐ILDs, highlighting that imaging findings should always be interpreted within their clinical context [26].

Emerging technologies such as photon‐counting CT and molecular imaging techniques may represent the next frontier, enabling earlier detection of active fibrogenesis and more accurate assessment of disease progression.

Photon‐counting CT is an emerging imaging technology that differs from conventional CT by using photon‐counting detectors, which directly count and measure the energy of individual X‐ray photons rather than integrating their total energy [27]. This technology provides higher spatial resolution, improved image quality with reduced electronic noise, and lower radiation exposure compared with conventional HRCT, potentially facilitating the detection of subtle fibrotic abnormalities.

Importantly, recent evidence suggests that these technical advantages may also enhance the performance of quantitative imaging algorithms, with CALIPER‐based lung texture analysis showing improved accuracy for detecting ILD features on PCCT despite being originally trained on conventional CT datasets [28].

Nevertheless, the high cost of this technology, limited availability, and the lack of standardized acquisition protocols and validated post‐processing pipelines currently restrict the widespread clinical implementation of PCCT.

Beyond structural imaging, fibroblast activation protein inhibitor positron emission tomography (FAPI‐PET) has recently emerged as a promising molecular imaging technique for ILDs. Unlike HRCT, which depicts predominantly established structural abnormalities, FAPI‐PET targets fibroblast activation protein (FAP), a membrane‐bound serine protease selectively overexpressed by activated fibroblasts during active fibrogenesis. Consequently, tracer uptake reflects ongoing fibroblast activity rather than irreversible fibrosis alone. Recent systematic reviews have shown that pulmonary FAPI uptake strongly correlates with fibrotic abnormalities on HRCT, baseline pulmonary function impairment, and histological FAP expression. Furthermore, increased baseline FAPI uptake has been associated with subsequent disease progression, suggesting a potential role in early risk stratification, monitoring of fibrotic activity, and treatment response. Although these preliminary findings are highly encouraging, prospective multicentre studies are still required before FAPI‐PET can be incorporated into routine clinical practice [29, 30, 31].

## 6 Therapy and Persistent Unmet Needs

**Summary**

- Treatment strategies are tailored to the underlying ILD subtype and disease behaviour.
- Nerandomilast and inhaled treprostinil are expanding antifibrotic treatment options.
- Earlier intervention, combination therapy, and biomarker‐guided treatment are emerging therapeutic priorities.

ILD treatment depends on the underlying disease. While antifibrotic therapy is the cornerstone of IPF management, treatment of non‐IPF ILDs is tailored according to the specific subtype. These principles have recently been formalized in the 2025 ERS/EULAR Clinical Practice Guidelines for CTD‐ILD [11]. This document provides the first comprehensive multidisciplinary recommendations covering screening, diagnosis, monitoring and treatment of CTD‐associated ILDs, emphasizing individualized management according to the underlying rheumatic disease and the pattern of lung involvement [11].

Steroids and immunosuppressive therapies constitute the mainstay of treatment for several immune‐mediated ILDs, particularly CTD‐ILDs and sarcoidosis. In contrast, identification and avoidance of the inciting antigen remain crucial in both fibrotic and non‐fibrotic HP [32, 33].

Among immunosuppressive agents, mycophenolate mofetil has become the preferred first‐line steroid‐sparing therapy for most CTD‐ILDs because of its favourable efficacy and tolerability profile, while rituximab is increasingly used in refractory or progressive disease, particularly in SSc‐ILD [34, 35, 36, 37]. Regarding pulmonary sarcoidosis, the 2025 PREDMETH trial demonstrated the non‐inferiority of methotrexate versus prednisone as first‐line treatment, supporting methotrexate as a potential steroid‐sparing first‐line option in selected patients [38].

However, the evidence supporting the broader use of immunosuppression across fibrotic non‐IPF ILDs remains limited and heterogeneous. In a recent multicentre retrospective study using a clone‐censor‐weighting approach to emulate a target trial, initiation of mycophenolate mofetil or azathioprine was not associated with improved three‐year transplant‐free survival or more favourable lung function trajectories in patients with non‐IPF idiopathic interstitial pneumonias, fibrotic hypersensitivity pneumonitis, or CTD‐ILD. These findings challenge the assumption that immunosuppression is uniformly beneficial across fibrotic ILD subtypes and reinforce the need for more precise treatment stratification and prospective randomized trials [39].

In fibrosing non‐IPF ILDs, antifibrotic therapy is currently recommended once a progressive phenotype has been established according to international guidelines [3], reflecting the concept that treatment should target both the underlying disease and the progressive fibrotic process.

For more than a decade, pirfenidone and nintedanib represented the only approved antifibrotic therapies for IPF. Both reduce the rate of lung function decline without halting disease progression and are frequently associated with adverse events requiring dose reduction or treatment discontinuation.

After several unsuccessful randomized controlled trials [40, 41, 42], nerandomilast and inhaled treprostinil have brought renewed optimism to the therapeutic landscape of IPF and PPF.

Nerandomilast is an oral preferential inhibitor of phosphodiesterase‐4B with antifibrotic and immunomodulatory properties. The phase 3 FIBRONEER‐IPF and FIBRONEER‐ILD trials demonstrated that nerandomilast significantly reduced FVC decline both as monotherapy and in combination with background antifibrotic therapy, while maintaining a favourable safety profile. However, the interaction between pirfenidone and nerandomilast reduces nerandomilast plasma concentration by at least 50%, resulting in subtherapeutic drug exposure with the 9 mg twice‐daily regimen and limiting the use of this combination [43, 44].

The unexpected preservation of FVC observed in the INCREASE trial provided the rationale for investigating inhaled treprostinil as an antifibrotic therapy in IPF [45, 46]. In the TETON‐2 trial, inhaled treprostinil reduced the rate of FVC decline compared with placebo, regardless of background antifibrotic therapy [47].

Together, these trials support the feasibility of combination antifibrotic therapy. Nevertheless, several questions remain unanswered, including the optimal timing of combination treatment, potential pharmacokinetic interactions, cumulative toxicity, and identification of patients most likely to benefit from dual therapy.

Interestingly, subgroup analyses from TETON‐2 suggested a smaller treatment effect in patients receiving background antifibrotic therapy, although these findings require cautious interpretation and prospective confirmation [47].

There is a need for deeper study of drug interactions and caution for potentially additive side effects. TETON‐PPF is an RCT currently ongoing to investigate the efficacy and safety of inhaled Treprostinil in patients with PPF (NCT05943535).

Other promising compounds currently under clinical investigation include admilparant, a selective lysophosphatidic acid receptor 1 (LPA1) antagonist [48, 49] and rentosertib, a first‐in‐class AI‐generated small‐molecule inhibitor of TNIK, a first‐in‐class target in idiopathic pulmonary fibrosis (IPF) discovered using generative AI [50].

Although the therapeutic landscape is gradually expanding, important unmet needs remain. Currently available therapies slow—but do not halt or reverse—fibrosis; their impact on respiratory symptoms and quality of life is limited, and reliable biomarkers to guide treatment selection and monitor response are still lacking. Future strategies will likely rely on earlier intervention, combination therapy targeting complementary pathogenic pathways, and precision medicine approaches integrating molecular and imaging biomarkers. Ongoing trials exploring treatment before overt disease progression, including patients with ILA (NCT07201922) and non‐progressive fibrosing ILDs (NCT07540988), may further redefine the therapeutic paradigm.

## 7 Conclusion

The management of ILDs is entering a new era, driven by advances in early diagnosis, molecular characterization, imaging, and targeted therapies. Although important unmet needs remain, the integration of multidisciplinary expertise with clinical, radiological, and molecular information will be essential to identify the right patient at the right stage of disease for the right therapeutic strategy, ultimately aiming to intervene earlier and improve long‐term outcomes.

## Author Contributions

**Tonia Magrì:** conceptualization, methodology, writing – original draft, writing – review and editing, investigation. **Luca Richeldi:** conceptualization, writing – review and editing, methodology, supervision.

## Funding

The authors have nothing to report.

## Conflicts of Interest

The authors declare no conflicts of interest.

## Data Availability

Data sharing not applicable to this article as no datasets were generated or analysed during the current study.
